# A Novel Protocol for Culturing Polarized Proximal Tubular Epithelial Cells from Kidney Biopsies: Enhancing Platforms for Drug Excretion and Nephrotoxicity Studies

**DOI:** 10.3390/jox15020052

**Published:** 2025-04-01

**Authors:** Tadej Petreski, Lidija Gradišnik, Luka Varda, Polona Kovačič, Jurij Dolenšek, Andraž Stožer, Sebastjan Bevc, Uroš Maver

**Affiliations:** 1 Department of Nephrology, University Medical Centre Maribor, Ljubljanska ulica 5, 2000 Maribor, Slovenia; tadej.petreski@student.um.si; 2 Institute of Biomedical Sciences, Faculty of Medicine, University of Maribor, Taborska ulica 8, 2000 Maribor, Slovenia; lidija.gradisnik@um.si; 3 Department of Dialysis, University Medical Centre Maribor, Ljubljanska ulica 5, 2000 Maribor, Slovenia; luka.varda@student.um.si; 4 Department of Physiology, Faculty of Medicine, University of Maribor, Taborska ulica 8, 2000 Maribor, Slovenia; polona.kovacic1@um.si (P.K.); jurij.dolensek@um.si (J.D.); andraz.stozer@um.si (A.S.); 5 Department of Biology, Faculty of Natural Sciences and Mathematics, University of Maribor, Koroška cesta 160, 2000 Maribor, Slovenia; 6 Department of Pharmacology, Faculty of Medicine, University of Maribor, Taborska ulica 8, 2000 Maribor, Slovenia; 7 Department of Internal Medicine, Faculty of Medicine, University of Maribor, Taborska ulica 8, 2000 Maribor, Slovenia

**Keywords:** cell separation, epithelial cells, large-core needle biopsy, phenotype, renal excretion, toxicity

## Abstract

The kidneys are integral to homeostasis but are susceptible to nephrotoxic compounds. Proximal tubular epithelial cells (PTECs) mediate drug metabolism and transport and are widely used in preclinical studies. However, commercial PTECs are limited in availability and physiological relevance. This study aimed to develop a novel, reliable protocol for isolating and culturing PTECs from human kidney biopsies. Primary PTECs were isolated from kidney biopsies of two patients (MFUM-RPTEC-1 and MFUM-RPTEC-2). Their morphology, population doubling time, transepithelial electrical resistance (TEER), and phenotypic markers were evaluated. Polarization and transporter expression were analyzed using cells cultured on Transwell inserts. Colonies formed within 24–48 h, with confluence reached by 8–10 days and dome (hemicyst) formation by day 13. TEER values peaked at 190 Ω/cm^2^ after 7–14 days, confirming tight junction formation. Immunostaining identified characteristic markers (e.g., SGLT2, OAT1/3, OCT2, P-gp, MRP4, MATE1, N-cadherin, ZO-1, CK-18). Cells cultured on Transwell plates exhibited native polarization, expressing transporters crucial for drug excretion on apical and basolateral surfaces. We present two robust protocols for isolating and characterizing PTECs, offering a scalable method to obtain functional, polarized cells from scarce biopsy material. The isolated PTECs, therefore, present a valuable platform for preclinical studies, especially for drug excretion testing through the expressed transporters. Drug competition for these transporters during tubular secretion is also a common cause of nephrotoxicity.

## 1. Introduction

Chronic kidney disease (CKD) affects about 15% of US adults [1]. Researchers are developing new kidney replacement therapies (KRTs), such as renal assist devices and implantable bioartificial kidneys [2,3,4,5]. Several studies exploit in vitro cell isolation and culture, with PTECs being among the most studied cells for such use [2,3,5]. However, protocols for isolating other kidney cells have also been described [6]. Newer methods try to generate in vitro PTECs from pluripotent or mesenchymal cells [7].

Acute kidney injury (AKI), defined by a sudden decrease in glomerular filtration rate (GFR), has various causes and affects approximately 5–10% of hospitalized patients [8,9]. An observational study by Iavecchia et al. showed that half of AKI episodes during hospitalization were drug-related [10]. Since most of the related mechanistic studies were performed in experimental laboratory animals, it is worrisome that meta-analyses have shown that animal tests have correctly predicted human responses in no more than 50% of experiments [11,12]. Several in vitro models using primary human PTECs were developed to upgrade the reliability of mechanistic studies—these range from two-dimensional (2D) models to multicellular three-dimensional (3D) cultures like PTEC-on-a-chip [13,14].

PTECs are prime candidates for CKD-related and nephrotoxicity in vitro studies, as they are responsible for the reabsorption, secretion, and metabolism of several xenobiotics [15]. For active tubular secretion, PTECs take up drugs from blood on their basolateral side (predominantly by organic cation transporters (OCT) 2 and organic anion transporters (OAT) 1 and 3) and secrete these into the urine via the apical transporters (e.g., P-glycoprotein (P-gp), multidrug resistance-associated proteins (MRP) 2 and 4, multidrug and toxin extrusion proteins (MATE) 1 and 2-k, and breast cancer resistance protein (BCRP)) [16].

Limited performance of inhibition of apical transporters (e.g., through drugs or drug-drug interactions) results in substance accumulation inside the PTEC, leading to nephrotoxicity. Drug–drug interactions increase with polypharmacy and are estimated to be clinically relevant in ~20% [15,17]. Therefore, nephrotoxicity studies in relevant models are critical to ensure patient safety. Commercially available cell lines (e.g., human kidney 2 (HK-2)) often lack necessary transporters [18] and/or a suitable cell morphology and polarization [19].

PTECs are tall cuboidal epithelial cells that exhibit a cobblestone appearance and show the formation of domes (hemicysts) in culture. Hemicyst formation indicates functional trans-epithelial fluid transport and is present around day 20 when PTECs are grown on a basement membrane substrate [20,21]. PTECs are phenotypically characterized by the presence of several markers (e.g., aquaporin 1, N-cadherin, CD10, CD13, lotus lectin) and functionally characterized by the expression of brush border enzymes (e.g., gamma-glutamyl transferase (GGT), alanine aminopeptidase (AAP), lactate dehydrogenase (LDH)), drug transporters (e.g., OAT1, OAT3, OCT2, P-gp), and metabolic enzyme activity (e.g., cytochromes p450 (CYP) 3A4, CYP2D6), as well as by their cellular energetics and redox status [6]. Aquaporin 1 is a marker constitutively expressed in the proximal tubule and descending limb of Henley’s loop and is not present in the distal tubule. Its importance lies mainly in fluid reabsorption. CD13 or aminopeptidase M is a proximal brush border membrane protein exclusively expressed in PTECs in vivo [22]. N-cadherin is a calcium-dependent cell adhesion molecule and is the predominant cadherin in the proximal tubule, which is essentially absent in other nephron segments [23].

To date, only a few protocols for establishing a primary PTEC culture have been described [24,25,26,27,28]. One of the first protocols for PTEC isolation was developed in 1984 by Detrisac et al. using cadaveric kidneys and nephrectomy specimens [24]. Only two studies (from 1989 [29] and 1991 [30]) have reported PTEC isolation protocols using tissue acquired via large-needle kidney biopsy; yet neither study focuses on PTEC isolation and systematic characterization. Therefore, their described protocols have minimal potential for reproduction. The Section 4. Discussion compares our novel optimized protocol with the limitations of the abovementioned studies.

Given the need for a more suitable isolation protocol, we aimed to develop optimized protocols for culturing polarized PTECs from kidney biopsies, suitable for drug excretion and nephrotoxicity studies. The resulting PTEC lines, MFUM-RPTEC-1 and MFUM-RPTEC-2, were systematically characterized using several markers, morphological analysis, TEER measurement, and population doubling time (PDT) measurements. To the best of our knowledge, no previous study has reported confirmation of as many markers in a single study nor performed a detailed morphology analysis accompanied by other support measurements related to cell growth. Furthermore, no prior study has thoroughly characterized the isolated PTEC in conjunction with reporting an optimized/novel isolation protocol. We finish the study with proof of PTEC polarization on Transwell plates, confirming their potential for preclinical studies.

## 2. Materials and Methods

### 2.1. Materials

If not stated otherwise, all used materials and chemicals were laboratory grade and purchased from Thermo Fisher, Hessen, Germany (all cell media), Abcam, UK (all antibodies), and Sigma-Aldrich, Darmstadt, Germany (all other chemicals and reagents). All labware and chemicals used were sterilized for specific parts of the isolation process and cultivation using standard sterilization procedures.

### 2.2. Sample Origin and Procedure

Two kidney samples were obtained during regular, diagnostic large-needle biopsies. The first sample was from a patient with immunotactoid glomerulopathy with monoclonal deposits of IgG kappa, and the second sample was from a patient with IgA glomerulonephropathy. A more detailed patient description and histopathology report are available in Supplement S1.

The study was conducted per the Declaration of Helsinki and its subsequent amendments and was approved by the Republic of Slovenia National Medical Ethics Committee (Nr.: 0120-429/2021/7). The patients’ written consents were obtained.

On site of the performed biopsy, the kidney samples, about 5 mm long and 1 mm wide, were transferred into a preprepared 15 mL falcon tube filled with Advanced Dulbecco’s Modified Eagle Medium/Nutrient Mixture F-12 (Advanced DMEM/F12, Gibco), supplemented with 0.1 mg/mL streptomycin, 100 IU/mL penicillin, and 2 mM L-glutamine, and transferred to the Institute of Biomedical Sciences at the Faculty of Medicine, University of Maribor, Slovenia.

### 2.3. PTEC Isolation

**Figure 1:**
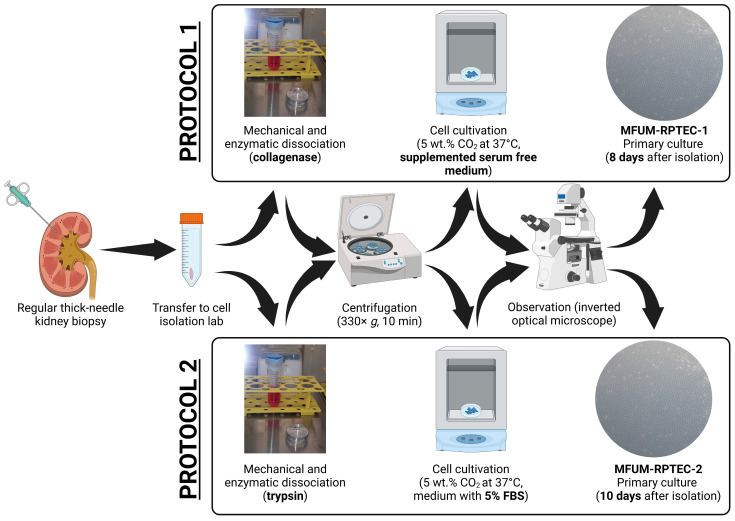
PTEC isolation from a kidney biopsy sample in a short overview of the most essential preparation steps.

Figure 1 schematically depicts an overview of the procedure steps for both used protocols. The protocols are briefly described below.

**Protocol 1**: In the cell isolation laboratory, the first tissue sample was washed with PBS, put into 1 mL 0.2% collagenase type 1 (Sigma), cut into smaller bits with a scalpel, and incubated for 1 h in a controlled atmosphere with 5 wt.% CO_2_ at 37 °C with occasional stirring (CO_2_ Incubator MCO-19AICUVH-PE; Panasonic, Tokyo, Japan). Next, the cell suspension was added to 4 mL of Advanced DMEM/F12. The suspension was transferred to a 15 mL falcon tube and centrifuged at 330× *g* for 10 min (Centrifuge 5804 R; Eppendorf, Hamburg, Germany). The supernatant was carefully discarded, and the cell pellet was re-suspended in a serum-free growth medium comprised of Advanced DMEM/F12 supplemented with Insulin–Transferrin–Selenium (ITS 100×, Gibco), epidermal growth factor (EGF, 10 ng/mL, Thermo Fischer), and hydrocortisone (36 ng/mL, Sigma) and seeded on 25 cm^2^ flasks, which were incubated in a controlled atmosphere with 5 wt.% CO_2_ at 37 °C. Growing cells were regularly observed with an Axiovert 40 inverted optical microscope (Zeiss, Oberkochen, Germany). The serum-free growth medium was changed every three days. The cells obtained based on protocol 1 were termed MFUM-RPTEC-1.

**Protocol 2**: In the cell isolation laboratory, the second tissue sample was washed with PBS, put into 1.5 mL 0.25% trypsin/EDTA (Sigma), cut into smaller bits with a scalpel, and incubated for 1 h in a controlled atmosphere with 5 wt.% CO_2_ at 37 °C with occasional stirring (CO_2_ Incubator MCO-19AICUVH-PE; Panasonic, Tokyo, Japan). Next, the cell suspension was added to 8.5 mL of DMEM/F12. The suspension was transferred to a 15 mL falcon tube and centrifuged at 400× *g* for 10 min (Centrifuge 5804 R; Eppendorf, Hamburg, Germany). The supernatant was carefully discarded, and the cell pellet was resuspended in Advanced DMEM/F12 medium supplemented with 5% fetal bovine serum (FBS) and seeded on 25 cm^2^ flasks, which were incubated in a controlled atmosphere with 5 wt.% CO_2_ at 37 °C. Growing cells were regularly observed with the inverted optical microscope. The medium was changed every three days. The cells obtained based on protocol 2 were termed MFUM-RPTEC-2.

Further measurements were conducted in various cell passages. Hemicysts were observed in the primary culture when following protocol 1 and in the second passage when following protocol 2. Transepithelial electrical resistance (TEER) was measured at different stages of the isolation and culture. Population doubling time (PDT) was measured only for the second passage, where the cells exhibited a clear morphological homogeneity without any remnants of other cell types. The protocols used for TEER and PDT measurement are described in Supplement S2 (additional micrographs showing the confluence reached before TEER measurements are shown in Supplemental Figure S1). Other characterization procedures (e.g., morphology evaluation, immunocytochemistry, etc.) are described below.

### 2.4. Cell Culture Freezing and Thawing Protocol

After reaching confluence, the cells were enzymatically detached using 0.25% trypsin/EDTA at 37 °C until fully dissociated from the culture surface. The resulting cell suspension was neutralized with an equal amount of fresh cell medium, transferred to a conical tube, and centrifuged at 330× *g* for 5 min. The supernatant was discarded, and the cell pellet was gently resuspended in a chilled freezing medium of 90% FBS and 10% dimethyl sulfoxide (DMSO). The cell pellet was placed into Mr. Frosty™ freezing containers (Thermo Fisher Scientific) and frozen to −80 °C. After 24 h at −80 °C, the vials were transferred to liquid nitrogen (−196 °C) for long-term storage.

Cryogenic vials were removed from liquid nitrogen to recover the cells and immediately placed in a 37 °C water bath, ensuring rapid thawing to preserve cell viability. Once thawed, the cell suspension was transferred into a warm culture medium and centrifuged under the same conditions described above to remove any residual DMSO. The cell pellet was then resuspended in a fresh culture medium and plated. Cells were considered to have advanced by one additional passage upon thawing, recognizing the combined effects of enzymatic detachment, cryopreservation, and reseeding on their overall passage history.

### 2.5. Characterization

#### 2.5.1. Cell Culture Protocol for Characterization

Due to the known possible PTEC dedifferentiation in culture [31,32], most characterization methods were conducted on either the second and/or third passage of MFUM-RPTEC-1 and MFUM-RPTEC-2. We aimed to show the stability of their phenotypic properties up to the third passage (enabling preclinical experiments such as drug excretion and nephrotoxicity studies).

**Figure 2:**
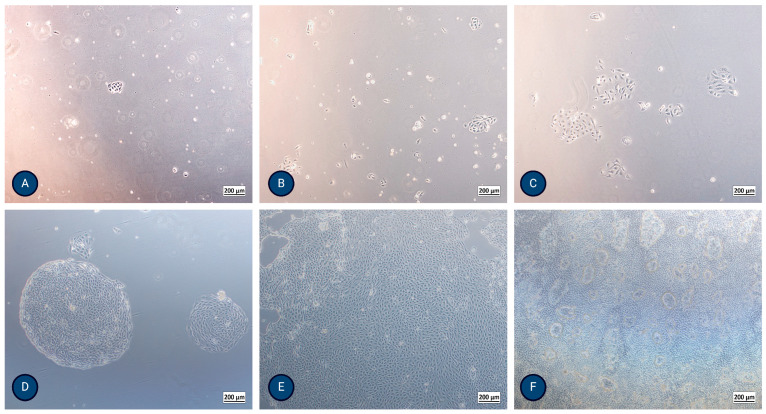
Micrographs of typical PTEC growth using inverted optical microscopy after (**A**) 24 h, (**B**) 48 h, (**C**) 72 h, (**D**) 5 days, (**E**) 8 days, and (**F**) 13 days, where formation of domes (hemicysts) is evident. The magnification of the images shown is 50× (Axiovert 40, Zeiss, Germany).

**Both protocols**: Cells were seeded, cultured in 25 cm^2^ flasks, and incubated in a controlled atmosphere with 5 wt.% CO_2_ at 37 °C. We used the media described above. After reaching confluence and/or forming hemicysts, as shown in Figure 2F, they were enzymatically treated using 0.25% trypsin/EDTA and frozen using 90% FBS and 10% dimethyl sulfoxide (DMSO). After thawing, the cells were considered one passage higher than when frozen.

Most micrographs were taken using an EVOS FL Cell Imaging System (Thermo Fisher Scientific, Waltham, MA, USA).

#### 2.5.2. Immunocytochemistry

For immunochemistry, we used round glass slides with 12 mm diameter, previously sterilized with 70% ethanol, placed on the bottom of wells in a P24 plate (in triplicate for phalloidin and duplicate for other markers). Isolated cells from the second passage at a density of 50,000 cells/well were placed on each glass slide and incubated at 37 °C, 5% CO_2_, for three days. The growth medium was removed, and the cells were washed with PBS. Cell fixation was performed using the Fixation Solution (Millipore, Billerica, MA, USA) for 15 min at room temperature, followed by washing the cells three times with ice-cold PBS for 5 min.

Critical PTEC markers on the apical (sodium–glucose cotransporter 2 (SGLT2), P-gp, MATE1, and N-cadherin) or basolateral (OAT1 and OAT3, OCT2, N-cadherin, MRP4) membrane were determined. Additionally, we stained the cells with epithelial lineage markers CK-18 and ZO-1. Cells’ general morphology was evaluated by staining the cytoskeleton (actin) using Phalloidin iFluor 555 Reagent (Abcam, Cambridge, UK) and nucleus using a mounting medium with 40,6-diamidino-2-phenylindole (DAPI; Sigma-Aldrich, Munich, Germany). Detailed descriptions of respective methods are part of Supplement S3.

#### 2.5.3. Cell Polarisation

To evaluate the cell polarization, crucial for PTEC native function in the nephron, we have seeded and grown MFUM-RPTEC-1 and MFUM-RPTEC-2 on Transwell inserts (with a polycarbonate membrane (Corning, Glendale, Arizona, USA)) at a density of 40.000 cells/well. The cells were stained for two apical (P-gp and MATE-1), two basolateral (OCT-2 and MRP-4), and two epithelial lineage markers, CK-18 and ZO-1. Fixation of cells was performed using 4% formaldehyde for 10 min at room temperature, followed by washing the cells three times with PBS for 5 min. Further details about the method are part of Supplement S4. A LEICA SP8 Stellaris upright confocal microscope (Leica Microsystems, Germany) was used for cell polarization evaluation. The protocol for confocal microscopy is described in Supplement S5.

## 3. Results

### 3.1. Isolation of Proximal Tubular Epithelial Cells

The morphology and proliferation of the isolated PTECs were regularly observed using inverted optical microscopy (Figure 2) and actin staining. After thawing, the cells showed a 98–100% survival rate.

**MFUM-RPTEC-1 isolation (protocol 1)**: The isolated cells formed the first colonies after 24 h. They exhibited a polygonal, cobblestone appearance, were homogenous, reached confluence after 8 days, and showed dome (hemicyst) formation after 13 days (Figure 2F and Supplemental Figure S2). TEER reached a maximum value of 170 Ω/cm^2^ after 7–9 days, showing a high integrity of the monolayer. The population doubling time of the second passage was 23.6 h.

**MFUM-RPTEC-2 isolation (protocol 2)**: The isolated cells formed the first colonies after 48 h. They exhibited a polygonal, cobblestone appearance, were homogenous, and reached confluence after 10 days. Dome (hemicyst) formation was seen in the second passage after 7 days. TEER reached a maximum value of 192 Ω/cm^2^ after 7–9 days. The population doubling time of the second passage was 29.7 h.

Table 1 compares the main characteristics of isolated cells in this study with the only two PTE cultures from biopsies reported in the literature.

### 3.2. Immunocytochemistry

**Figure 3:**
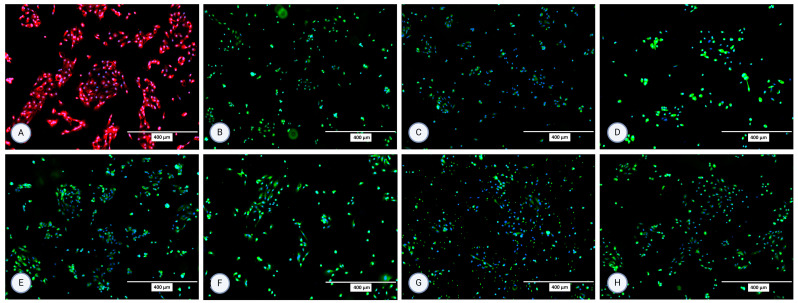
Micrographs of the stained samples in the second passage following protocol 1 for (**A**) P-gp, (**B**) OCT2, (**C**) N-cadherin, (**D**) SGLT-2, (**E**) MRP4, (**F**) MATE1, (**G**) OAT1, and (**H**) OAT3. A mounting medium with DAPI was used to stain the nuclei for all samples. The fluorescence signal from the specific markers, particularly those labeled in green, is highly intense, which results in the DAPI signal appearing less prominent or overshadowed in the images. The magnification of all shown images is 10× (according to the manufacturer’s microscope specifications, EVOS FL Cell Imaging System, Thermo Fisher Scientific, USA). In some micrographs, the intensity of specific marker staining partially overshadowed the DAPI signal; however, this does not reflect an absence of nuclei or question the cell identity.

**Figure 4:**
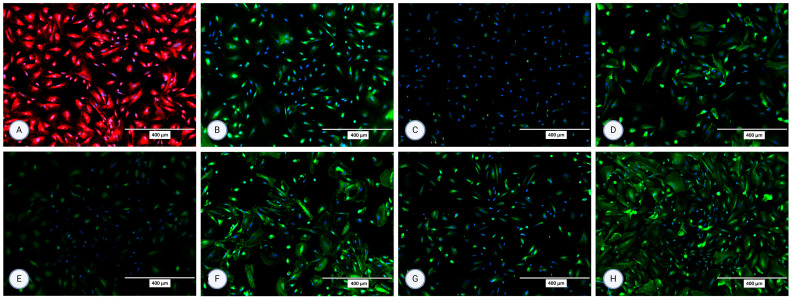
Micrographs of the stained samples in the second passage following protocol 2 for (**A**) P-gp, (**B**) OCT2, (**C**) N-cadherin, (**D**) SGLT-2, (**E**) MRP4, (**F**) MATE1, (**G**) OAT1, and (**H**) OAT3. A mounting medium with DAPI was used to stain the nuclei for all samples. The fluorescence signal from the specific markers, particularly those labeled in green, is highly intense, which results in the DAPI signal appearing less prominent or overshadowed in the images. The magnification of all shown images is 10× (according to the manufacturer’s microscope specifications, EVOS FL Cell Imaging System, Thermo Fisher Scientific, USA). In some micrographs, the intensity of specific marker staining partially overshadowed the DAPI signal; however, this does not reflect an absence of nuclei or question the cell identity.

The isolated cells were characterized using immunocytochemistry for several surface proteins in the second and third passage. All staining was performed in (at least) two repetitions. For MFUM-RPTEC-1, the presence of all selected proteins (P-gp, OCT2, N-cadherin, SGLT2, MRP4, MATE1, OAT1, OAT3) was confirmed in the second passage (Figure 3). In the third passage, expression and signals for OCT2 and OAT1 were lost (Supplemental Figure S3). For MFUM-RPTEC-2, all proteins were confirmed in the second passage (Figure 4) and the third passage (Supplemental Figure S4). When stained for podocin, the isolated cells did not show any signal, which served as a negative control in that the isolated cells had no podocytes. No signal was seen in either the second or third passage (Supplemental Figure S5 shows the micrographs of podocin-stained cells in the third passage).

### 3.3. Cell Polarisation

**Figure 5:**
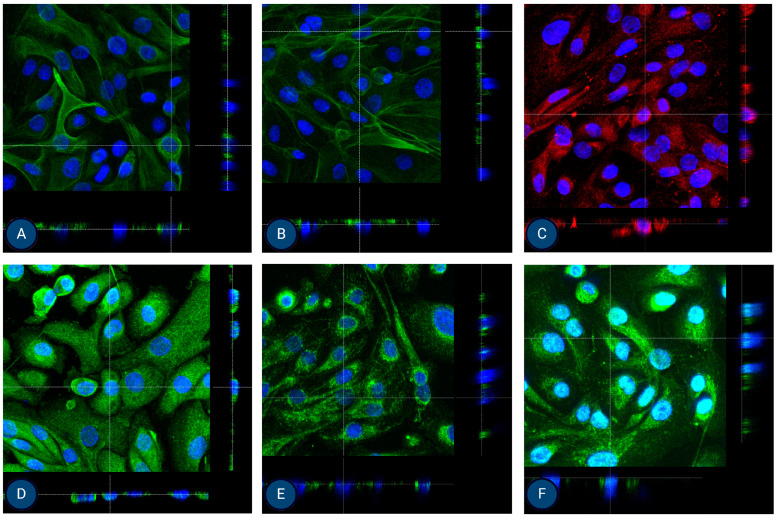
Orthogonal images of stained MFUM-RPTEC-1 grown on inserts in the first passage following protocol 1 shown in green and red for (**A**) CK-18, (**B**) phalloidin, (**C**) P-gp, (**D**) MATE-1, (**E**) MRP4, and (**F**) OCT2. A mounting medium with DAPI was used to stain the nuclei (shown in blue) for all samples. The magnification of all images shown is 63× (according to the manufacturer’s microscope specifications, LEICA SP8 Stellaris upright confocal system, Leica Microsystems, Germany).

**Figure 6:**
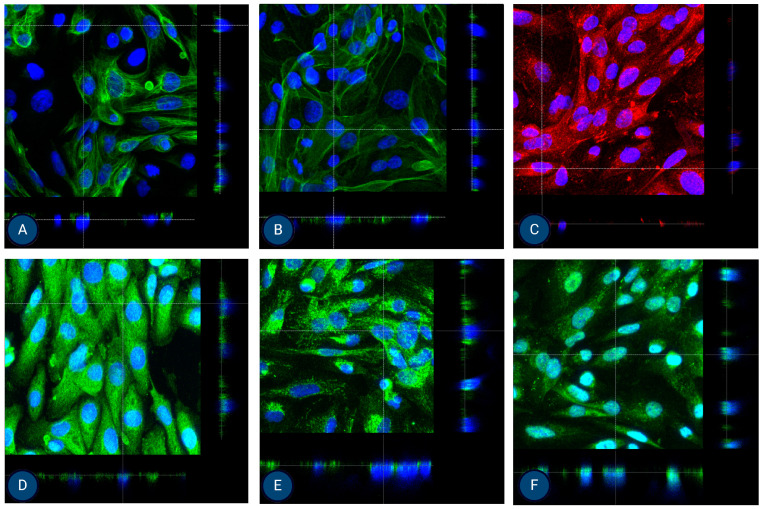
Orthogonal images of stained MFUM-RPTEC-2 grown on inserts in the first passage following protocol 2 shown in green and red for (**A**) CK-18, (**B**) phalloidin, (**C**) P-gp, (**D**) MATE-1, (**E**) MRP4, and (**F**) OCT2. A mounting medium with DAPI was used to stain the nuclei (shown in blue) for all samples. The magnification of all images shown is 63× (according to the manufacturer’s microscope specifications, LEICA SP8 Stellaris upright confocal system, Leica Microsystems, Germany).

After growing MFUM-RPTEC-1 and MFUM-RPTEC-2 on Tranwell inserts for 7–9 days, confluent cultures were obtained with high TEERs values, 170 Ω/cm^2^ and 192 Ω/cm^2^, respectively. At this time point, the cells were fixated on the inserts and stained for the chosen apical (P-gp and MATE-1), basolateral (OCT-2 and MRP-4), and epithelial lineage (CK-18 and ZO-1—shown in Supplementary Figure S8) markers, and for the cytoskeleton (phalloidin). Confocal microscopy confirmed the presence of all markers at their expected sites. Through this, a successful polarisation was shown for both MFUM-RPTEC-1 and MFUM-RPTEC-2, indirectly proving the suitability of both protocols (1 and 2) to yield viable and functional PTECs. The obtained micrographs are shown in Figure 5 (3D micrographs are shown in Supplemental Figure S6) for MFUM-RPTEC-1 and Figure 6 (3D micrographs are shown in Supplemental Figure S7) for MFUM-RPTEC-2.

## 4. Discussion

Our isolation approach, based on biopsy-derived samples, has several intrinsic advantages compared to full-kidney isolations. For example, it minimizes the presence of unwanted nephron segments in the tissue sample, reducing the need for additional purification steps [33]. To ensure the best conditions to isolate PTECs with high specificity, we performed regular media changes and morphological observations, followed by phenotypic characterization in the second and third passages, as well as functional validation, together indicating a homogeneous PTEC culture. As our cells exhibited a well-characterized PTEC phenotype with no podocyte contamination, we did not find it necessary to assess distal tubule markers such as ENaC.

Following kidney biopsy, we developed two novel and optimized cell isolation and culture protocols. These protocols differ in the enzymatic degradation method and cell culture medium composition (shown in Figure 1 and described in the Section 2). The first main difference between the two protocols is in the use of digestion enzymes, collagenase (protocol 1) and trypsin (protocol 2). Each digestion method presents specific advantages and limitations, which we have observed also in our previous cell isolation studies [34,35]. Trypsin enables faster dissociation of cells but is known to induce higher cell death due to its non-specific proteolytic activity [36,37]. To mitigate these effects, optimizing digestion time is often crucial. In contrast, collagenase digestion is more selective towards collagen fibers while preserving other extracellular matrix components, which often results in higher cell viability and isolation yields. However, it requires prolonged incubation times, which may, in turn, also impact cell viability and yield [38]. Studies have shown that lower concentrations of collagenase over extended periods can yield higher numbers of viable cells, whereas higher concentrations may require shorter incubation times to prevent cell damage [37].

Another considered optimization of the protocols was related to the use of FBS as a supplement to the cell media. We acknowledge that FBS promotes the proliferation of fibroblasts, which might affect the purity of the isolated proximal tubular epithelial cells (PTECs). This phenomenon is attributed to the growth factors present in FBS that stimulate fibroblast growth [39]. Despite this known issue, we can confirm that in neither of the protocols, has such a phenomenon occurred. One of our main goals was to preserve the desired PTEC phenotype to at least the third passage, which enables us to grow a sufficient number of cells for further experimentation [40]. During our chosen culturing conditions, no fibroblasts were observed; in addition, a high level of desired marker expression was shown for the second and third passages, respectively. This is in agreement with previous studies [41,42]. Based on these results, we cannot exclude that fibroblast overgrowth could occur in later passages, but for the given conditions, this did not cause any concerns in our protocols. Nonetheless, they both yield a PTEC culture with the same phenotypical and functional characteristics; they differ only slightly in their morphology and growth characteristics, which could have practical implications for developing functional models.

PTECs are critical for different preclinical studies, reflecting human kidney disease’s cellular and molecular environment [43]. When included in in vitro models, these cells help identify specific pathophysiological mechanisms of the kidney, enabling the development of targeted therapies that could be more effective and have fewer side effects [44]. In addition, using PTECs can improve the predictive accuracy of preclinical models, bridging the gap between laboratory research and clinical application in nephrology [45]. However, most studies that report using PTECs rely on either commercial cell lines or have isolated their cells from nephrectomy samples or post-mortem [46,47]. Although some reviews mention the possibility of isolation via large-needle biopsy, to our knowledge, none cite isolation protocols specifically designed for this [6,43,48,49]. One of the main limiting factors for this tissue source is the small number of cells contained in one biopsy core, which usually ranges between 40.000 and 75.000 cells [50]. One also cannot abide by the patient’s kidney disease diagnosis, which in our cases had a minimal impact on the tubular matrix with only 10 and 15% tubulointerstitial fibrosis. This could be the reason for the results achieved. However, we suspect predominantly healthy cells were isolated, as there is a positive selection bias when culturing cells [39,51].

Miltenburg et al. and Müller et al. reported PTEC isolation protocols from kidney biopsy samples. However, since the goals of these studies were very different from ensuring an efficient protocol for the isolation of phenotypically homogenous PTECs for preclinical model preparation, their protocols were also not directly applicable to our study. Both studies mentioned above aimed to investigate the cellular interactions between graft infiltrating cells and PTECs in kidneys undergoing rejections and to get more insight into the pathomechanism of kidney failure in patients with interstitial fibrosis, respectively [29,30]. Miltenburg et al. followed the protocol from Detrisac to culture PTECs, which had primarily been developed to isolate PTECs from nephrectomy specimens and faces challenges, such as a very heterogenous cell population surviving in culture, which makes it more difficult to isolate specific cells [52]. Conversely, Müller et al. grew a mixed primary culture, which they sorted using a fluorescence-activated cell sorter (FACS) with monoclonal antibodies. PTECs were then reseeded. This results in losing one passage, which limits the usefulness and leads to a smaller cell yield, as primary cultures of PTECs start losing their phenotype after the third passage [53].

Morphologically, PTECs and endothelial cells demonstrate a polygonal, cobblestone appearance and are subject to contact inhibition in culture [20,21,54]. They can be distinguished from mesangial cells that are elongated and stellate-shaped, not subject to contact inhibition, and grow in multilayers, similar to vascular smooth muscle cells [54]. Several days after reaching confluence, PTECs show dome/hemicyst formation, indicating active transport of solutes and water and cell-to-substrate interaction [55]. This was demonstrated by Detrisac et al. [24] and Miltenburg et al. [29] and is well documented in our work and depicted in Figure 2 and Supplemental Figure S2.

Miltenburg et al. and Müller et al. used characterization markers that prove the epithelial lineage of PTECs but may not be specific for their desired native phenotype nor truly show their functionality. This is also why protocols derived from nephrectomy specimens use several different characterization methods. Prasad et al. pointed out the importance of markers related to the PTECs’ function. They showed that six of the most abundant drug transporters in the kidney cortex are OCT2, OAT1, MATE1, SGLT2, OAT3, and P-gp [56]. OCT2 and SGLT2 transporters are PTEC specific, where OCT2 is located at the basolateral membrane of whole segments of the proximal tubule, and SGLT2 is found at the apical membrane of S1 and S2 segments of the proximal tubule [57,58]. Others are less specific but important for drug transport and, therefore, key to the tubular excretion function of PTECs [59,60,61,62,63]. N-cadherin is a non-specific cadherin in neural, muscle, and mesenchymal cells. However, PTECs have been shown to specifically express N-cadherin, which correlates with acute tubular necrosis in in vitro studies [64,65]. Especially for nephrotoxicity studies, PTECs should exhibit major classes of drug transporters such as SGLT-2, MRP4, OAT, OCT, and P-gp [66], which was proven for MFUM-RPTEC-1 and MFUM-RPTEC-2. Based on the obtained results, confirming the presence of all the most critical phenotypic and functional markers of PTEC in both isolated cell lines (MFUM-RPTEC-1 and MFUM-RPTEC-2), we believe in having prepared two optimized PTEC isolation protocols that enable the culturing of a high enough number of cells (with a stable phenotype until the third passage) that can be used for different preclinical studies (e.g., drug excretion and nephrotoxicity). Considering the base tissue from the biopsy (which presents an ideal and readily available “waste” source material for cell isolation) with a limited number of available cells for isolation, this seems an even more remarkable achievement. It is worth mentioning that both isolated cell lines, MFUM-RPTEC-1 and MFUM-RPTEC-2, besides the essential drug transporters, also express the PTEC-specific N-cadherin, while staining for podocin as a marker of podocytes was negative [67]. Our staining showed a stable phenotypic (e.g., N-cadherin, SGLT-2, etc.) and functional (e.g., drug transporters, like OCT2) marker profile in the second and third passages. The absence of podocin expression, together with the robust expression of multiple PTEC-specific markers, confirms the proximal tubule identity of the cultured cells. While fibroblast-specific markers were not included, the cells displayed typical epithelial morphology and clear PTEC-related marker expression, with no evidence of fibroblast overgrowth.

Another significant difference between previous studies and our own is the systematic approach to characterizing cell culture from the primary culture to the third passage. For example, the studies by Miltenburg et al. and Müller et al. did not report cultivation information, such as how fast the cells grow, i.e., when they reach confluence, and what their PDT is. They also did not confirm the epithelial formation through TEER measurement, a critical parameter in performing nephrotoxicity and transport studies. TEER is a non-invasive method to assess the barrier function of the monolayer, i.e., the tightness of the connections between cells. TEER values for human PTECs have usually stabilized at around 100 Ω/cm^2^ [68], while we demonstrated even higher values at 170 and 192 Ω/cm^2^ for MFUM-RPTEC-1 and MFUM-RPTEC-2, respectively. An additional important cultivation measure is PDT, a standardized measure to show how fast the cells grow. Protocols for PTEC isolation rarely report this value. However, some immortalized cell lines, such as the HK-2 line, report PDT values of 53.7 h (56), while our cells grew even faster at a PDT of 23.6 h following protocol 1 (for isolation of MFUM-RPTEC-1) and a PDT of 29.7 h following protocol 2 (for isolation of MFUM-RPTEC-2). Faster-growing cells enable the formation of confluent cultures before dedifferentiation, which might hinder effective experimentation.

Finally, given our goal to establish efficient PTEC isolation protocols for preclinical studies in AKI and CKD, we also performed cell polarization studies. In their native form, PTECs polarise to achieve efficient (and well-controlled) drug transport via basolateral and apical transporters. To this end, MFUM-RPTEC-1 and MFUM-RPTEC-2 were grown on Transwell inserts. After reaching confluence (approximately after 7–9 days), we fixed them. We stained the cells for epithelial lineage markers (CK-18 and ZO-1) as well as for specific apical (P-gp and MATE-1) and basolateral (OCT-2 and MRP-4) drug transporters. Confocal microscopy was used to analyze marker localization in the cell monolayer (Figure 5 and Figure 6). For this purpose, we chose orthogonal images, as they provide a cross-sectional view of the sample by slicing through the Z-stack. This approach allows for a clearer distinction between apical and basolateral marker localization, which can be challenging to interpret in traditional 3D views (these can be seen in the Supplementary document as Supplemental Figures S6 and S7).

ZO-1 appears as a thin apical band at cell–cell contacts in orthogonal images, marking the boundary between the apical and basolateral domains (see Supplemental Figure S8). Positively stained ZO-1, which is known to localize at cell–cell junctions, confirms the presence of tight junctions for both isolated cells. CK18 is distributed throughout the cell cytoplasm. In orthogonal views, it appears as a diffuse intracellular signal, without specific localization to the membrane. MRP-4 is primarily localized to the basolateral membrane. In orthogonal images, it is seen at the lower part of the cell, facing the basolateral side. OCT2 is also found on the basolateral membrane. When viewed orthogonally, it appears at the basolateral side, similar to MRP-4. MATE1 and PGP are localized to the apical membrane. In orthogonal images, they are positioned at the top of the cell, corresponding to the apical surface. Here, we need to stress that in one cell line, MATE-1 was correctly localized to the apical membrane (MFUM-RPTEC-1), while in the second, the marker appeared basolaterally (MFUM-RPTEC-2). We hypothesize that this discrepancy may reflect donor-specific differences in cellular phenotype and underlying pathology. The MFUM-RPTEC-2 cells, showing altered MATE-1 localization, were notably larger, which may indicate a shift in differentiation or transport function. As discussed in the supplementary materials, these cells were derived from a patient diagnosed with IgA nephropathy, characterized by 62.5% glomerulosclerosis and 15% interstitial fibrosis with tubular atrophy. Furthermore, there were signs of chronic uric acid nephropathy and benign hypertensive nephrosclerosis. It is plausible that chronic injury, inflammation, or fibrosis may have influenced transporter expression and localization. Previous studies have reported altered MATE-1 localization in models of renal injury and fibrosis [69,70], suggesting that pathological conditions can affect the membrane trafficking of solute carriers. Thus, while apical expression is the expected pattern in healthy PTEC, variations in MATE-1 localization may be a biologically relevant reflection of donor pathology, indicating that our protocols might be even able to preserve donor-specific pathological features. Of course, further studies will be necessary to confirm this.

Despite the latter, the correct membrane localization of these transporters highlights the suitability of our MFUM-RPTEC lines for in vitro drug excretion studies, including potential drug–drug interactions. For example, these cells can be used to study apical efflux via P-gp (using cyclosporine as the classical substrate) and MATE-1 (using metformin in the case of MFUM-RPTEC-1) or basolateral uptake and efflux via OCT-2 (modeled by cimetidine) and MRP-4 (using methotrexate). As these transporters take up many clinically relevant drugs and are key sites for drug–drug interactions, our isolated PTECs provide a robust system for preclinical investigation of renal excretory function and testing drug-drug interaction-related nephrotoxicity.

Nevertheless, our protocols and models have some limitations. 2D in vitro models are widely used owing to their simplicity and cost-effectiveness, making them particularly suitable for high-throughput screening. These models also offer high reproducibility and lend themselves readily to standardized assays. However, with duplication, they often lose key physiological and morphological characteristics, including transporter expression and essential cell–cell interactions, and the absence of an in vivo–like extracellular matrix and microfluidic environment further limits their predictive capability [71,72].

By contrast, 3D kidney-on-a-chip platforms more closely approximate the in vivo microenvironment, incorporating fluid shear stress, cell–cell interactions, and complex tubular architecture vital for accurate drug transport and nephrotoxicity assessments. These systems recreate dynamic mechanical forces and tissue structures that are highly relevant to renal physiology and pathology. Nonetheless, the complexity of such platforms can lead to higher costs and technical challenges, and scaling them for high-throughput screening remains problematic [73,74].

Induced pluripotent stem cell (iPSC)-derived kidney models add another dimension of physiological relevance by providing human-specific responses, particularly valuable for personalized medicine and disease modeling. These models can differentiate into multiple renal cell types, offering a more comprehensive representation of kidney function and pathology. However, current protocols often generate only partially mature organoids with occasional off-target lineages, limiting their functional relevance. Moreover, successfully generating and maintaining iPSC-derived kidney models require specialized expertise and resources, increasing complexity and cost [75,76].

It has also to be stressed that our results indicate a sufficient cell yield for all the performed (and targeted) experiments. Since PTECs proliferated efficiently up to passage 3 (P3) without limitations requiring further optimization, further experimentation with either of the newly developed cell lines is ensured. A possible issue with similar cell lines could also be senescence [77]. The latter was not observed in our study, as cells maintained epithelial morphology, proliferative capacity, and functional polarization, aligning with prior studies on primary PTEC cultures [78]. Additionally, phenotypic analysis and functional validation confirmed stable expression of PTEC-specific markers, demonstrating that de-differentiation did not occur within the tested timeframe.

To conclude, we present robust protocols for isolating and characterizing PTECs, offering a scalable method to obtain functional, polarized cells from limited biopsy material. The resulting cultures, which maintain key transporter expression, provide a valuable platform for preclinical studies to elucidate drug excretion and nephrotoxic mechanisms. This in vitro model facilitates targeted investigations of drug competition during tubular secretion, a frequently overlooked factor in nephrotoxicity. Although our current PTEC model was not explicitly designed for high-throughput screening (HTS), future adaptations could enable its use in such applications. Scaling up the system by culturing multiple PTEC isolates in parallel, for example, using multi-well plates with Transwell inserts could facilitate simultaneous compound screening.

## Figures and Tables

**Table 1:** Comparison between our own and published PTEC isolation protocols following kidney biopsy and cell line characteristics.

**Authors, year**	Miltenburg et al., 1989 [29]	Müller et al., 1991 [30]	This study, 2024
**Target cells**	PTEC, GIC	PTEC, renal fibroblasts	PTEC
**Source**	renal allograft	severe glomerulonephritis	immune complex glomerulonephritis and IgA nephropathy
**Culture medium**	DMEM/F12	RPMI 1640	Advanced DMEM/F12
**Supplements**	ITS, hydrocortisone, T3, EGF	20% FCS	Protocol 1: ITS, hydrocortisone, EGF Protocol 2: 5% FBS
**Characterization**	light microscopy, immunofluorescence	light microscopy, FACS	light microscopy, immunofluorescence, confocal microscopy
**Morphology**	as in protocol of Detrisac, “domes”	light microscopical criteria for epithelial cells	cobblestone appearance and hemicyst (“domes”) formation
**Immunofluorescence** (+: present; -: absent)	EMA+, ADBP (1071+, 1072+, 1079-, 1080-), MHC I+ and MHC II-	MHC I+, MHC II-	P-gp+, OCT2+, N-cadherin+, SGLT2+, MRP4+, MATE1+, OAT1/3+, ZO-1+, CK-18+
**Growth** (days until confluence)	NS	10–14	Protocol 1: 8 Protocol 2: 10
**PDT** (hours)	NS	NS	Protocol 1: 23.6 Protocol 2: 29.7
**TEER 24 h/48 h** (Ω/cm^2^)	NS	NS	Protocol 1: 120 ± 7/170 ± 8 Protocol 2: 192 ± 15/180 ± 10

NS—not specified; PTEC—proximal tubular epithelial cell; GIC—graft infiltrating cells; DMEM/F12—Dulbecco’s modified Eagle’s medium/Ham’s F12 medium; ITS—insulin, transferrin, selenium; T3—triiodothyronine; EGF—epidermal growth factor; FBS—fetal bovine serum; EMA—epithelial membrane antigen; ADBP—adenosine–deaminase binding protein; MHC—major histocompatibility complex; PDT—population doubling time; RPMI 1640—Roswell Park Memorial Institute 1640 medium; FCS—foetal calf serum; FACS—fluorescence-activated cell sorter; TEER—transepithelial electrical resistance; SGLT2—sodium–glucose cotransporter 2; MRP4—multidrug-resistant protein 4; OAT1/3—organic anionic transporter 1 and 3; OCT2—organic cationic transporter 2; P-gp—P-glycoprotein; MATE1—multidrug and toxin extrusion protein 1; ZO-1—zonula occludens 1; CK-18—cytokeratin 18.

## Data Availability

The original contributions presented in this study are included in the article/Supplementary Material. Further inquiries can be directed to the corresponding authors.

## Supplementary Materials

The following supporting information can be downloaded at: https://www.mdpi.com/article/10.3390/jox15020052/s1, Supplement S1. Sample origin description, Supplement S2. Population doubling time protocol, Supplement S3. Cytochemistry staining protocols, Supplement S4. Details about the cell polarisation evaluation, Supplement S5. Protocol for confocal microscopy used in polarisation experiments, Supplement S6. Supplementary Figures.
