# Supplementary material for: A Novel Protocol for Culturing Polarized Proximal Tubular Epithelial Cells from Kidney Biopsies: Enhancing Platforms for Drug Excretion and Nephrotoxicity Studies
Source: J Xenobiot. 2025 Apr 1;15(2):52. doi: 10.3390/jox15020052 (PMC12028765; doi:10.3390/jox15020052)
Supplement: Supplementary file 1 [file jox-15-00052-s001.zip › jox-3478667-supplementary.pdf]

SUPPLEMENTAL MATERIAL

A Novel Protocol for Culturing Polarized Proximal Tubular Epithelial Cells from Kidney Biopsies: Enhancing Platforms for Drug Excretion and Nephrotoxicity Studies

Tadej Petreski [1], Lidija Gradisňik [2], Luka Varda [3], Polona Kovačič [4], Jurij Dolenšek [4,5], Andraž Stožer [4], Sebastjan Bevc [1,6,7,*] and Uroš Maver [2,6,*]

[1] Department of Nephrology, University Medical Centre Maribor, Ljubljanska ulica 5, 2000 Maribor, Slovenia; tadej.petreski@student.um.si
[2] Institute of Biomedical Sciences, Faculty of Medicine, University of Maribor, Taborska ulica 8, 2000 Maribor, Slovenia; lidija.gradisnik@um.si
[3] Department of Dialysis, University Medical Centre Maribor, Ljubljanska ulica 5, 2000 Maribor, Slovenia; luka.varda@student.um.si
[4] Department of Physiology, Faculty of Medicine, University of Maribor, Taborska ulica 8, 2000 Maribor, Slove-nia; polona.kovacic1@um.si (P.K.); jurij.dolensek@um.si (J.D.); andraz.stozer@um.si (A.S.)
[5] Department of Biology, Faculty of Natural Sciences and Mathematics, University of Maribor, Koroška cesta 160, 2000 Maribor, Slovenia
[6] Department of Pharmacology, Faculty of Medicine, University of Maribor, Taborska ulica 8, 2000 Maribor, Slovenia
[7] Department of Internal Medicine, Faculty of Medicine, University of Maribor, Taborska ulica 8, 2000 Maribor, Slovenia
* Correspondence: sebastjan.bevc@ukc-mb.si (S.B.); uros.maver@um.si (U.M.)

Supplemental material table of contents

Supplement S1. Sample origin description

Supplement S2. TEER and population doubling time protocol

Supplement S3. Cytochemistry staining protocols

Supplement S4. Details about the cell polarisation evaluation

Supplement S5. Protocol for confocal microscopy used in polarisation experiments

Supplement S6. Figures

Supplement S1. Sample Origin Description

The first included sample was from a 60-year-old male with nephrotic range proteinuria, where standardly three cores were taken during biopsy to establish the diagnosis, while a part of the third core was used for our protocol. The patient had previously known CKD stage G3aA3, arterial hypertension, and dyslipidemia. The second sample was from a 61-year-old male taken to establish a diagnosis of proteinuria and hematuria. The patient had previously known CKD stage 3 and arterial hypertension.

The biopsies were performed at the University Medical Centre Maribor, Slovenia. The remaining 2.5 cores from the biopsies were sent to the Institute of Pathology at the Faculty of Medicine, University of Ljubljana, Slovenia, to be inspected and characterized according to established histopathological protocols for kidney biopsy samples. In the first case, the immunofluorescence showed monoclonal granular immune deposits IgG1 kappa: IgG1 (++), kappa (++), C3 (+++), and C1q (+++) in the glomerular capillary wall. The tubular matrix showed granular C3 (+) deposits in individual tubules. The final diagnosis for the patient's kidney disease was immunotactoid glomerulopathy with monoclonal deposits of IgG kappa with 20% glomerulosclerosis and up to 10% interstitial fibrosis and collapsible tubular atrophy and up to 10% interstitial inflammatory infiltrates. Signs of mild to moderate focal tubular damage were present. In the second case, immunofluorescence showed granular immune deposits of IgA (++), IgG (+), IgM ($\pm$), C3 (++), and fibrin/fibrinogen in the mesangium and segmentally in the glomerular capillary wall. The final diagnosis for the patient´s kidney disease was IgA glomerulonephropathy with 62.5% glomerulosclerosis and 15% interstitial fibrosis with tubular atrophy. The Oxford classification was M0, E0, S1, T0, C0. The tubules showed some calcification and one tubule showed uric acid tophi, which is indicative of chronic uric acid nephropathy. Additionally, benign hypertensive nephrosclerosis was present.

Supplement S2. Population Doubling Time Protocol and TEER Measurements

Isolated cells from the second passage were seeded onto a P6 plate at 300,000 cells / well density with 3 ml of serum-free growth medium in duplicate for each time point. After 24 h, 48 h, and 72 h, two wells were carefully emptied, respectively. Next, they were washed with 500 µl 0.25% trypsin/EDTA (Sigma, France) and incubated in 1mL of 0.25% trypsin/EDTA for 5 min. Next, 4mL of the serum-free growth medium was added, and the suspension was transferred to a 15 mL falcon tube and centrifuged at 330 x g for 5 min. The supernatant was carefully discarded, and the cell pellet was re-suspended in 1 mL of serum-free growth medium. The cells were counted using the 0.1% trypan blue exclusion test of cell viability.

For TEER measurements, MFUM-RPTEC-1 and MFUM-RPTEC-2 in the first and second passages, respectively, were seeded and cultured on Transwell inserts at a density of 100.000 cells / insert. We used 12 mm diameter inserts with a polyester membrane (Corning, USA). The TEER measurements were performed using a Millicell ERS-2 Voltohmmeter (Millipore, USA) to assess the barrier integrity of the isolated PTECs. After the confluence was reached (as stated in Table 1, after 8 days for MFUM-RPTEC-1 and after 10 days for MFUM-RPTEC-2) confirmed by an EVOS FL Cell Imaging System, (Thermo Fisher Scientific, USA)), TEER measurements were taken at 24 h and 48 h by placing the electrodes in both the apical and basolateral compartments, ensuring minimal disturbance to the cell layer. TEER values ($\Omega \cdot cm^2$) were calculated by subtracting the blank insert resistance from each measurement and multiplying by the membrane surface area. The reported values present an average of three measurements with standard errors for each of the two reported time points. During experimentation, the TEER measurements were also performed at various other time points after seeding (e.g., after 6, 8, and 10 days) for both

isolated cell lines (values varied from 120 to 192 Ω·cm². The optimal time frame for the start of the TEER measurement was found to be the one reported in Table 1 and above.

Supplement S3. Cytochemistry Staining Protocols

Supplement S3.1. Actin Staining

Following the protocol for immunocytochemistry, the working solution of the conjugated Phalloidin (1,000x Phalloidin stock solution in dimethyl sulfoxide DMSO (Abcam, UK), 1:1,000 dilution in PBS with 1% bovine serum albumin (BSA), and 0.1% Tween 20) was added. Incubation was performed for 90 min at room temperature and in a dark room. Rinsing was performed with PBS and was repeated three times. The final step was the addition of the Fluoroshield Mounting Medium with 4′,6-diamidino-2-phenylindole (DAPI). Micrographs were taken at the suitable wavelengths for respective dyes (excitation/emission: DAPI D 306/460 nm and Phalloidin D 556/574 nm).

Supplement S3.2. Staining for PTEC-Specific (Positive) Markers

Following the protocol for immunocytochemistry described above, the cells were incubated for 30 min with PBS, supplemented with 1% BSA and 0.1% solution of Tween 20 to block the non-specific binding of antibodies for primary antibodies. Incubation for 30 min with PBS supplemented only with 1% BSA was used for secondary antibodies. All incubations with the primary and conjugated antibodies were performed overnight at 4°C. Respective dilutions of the primary and conjugated antibodies were as follows:

1. P-gp: (Alexa Fluor 647) Anti-P-Glycoprotein antibody (Abcam, UK), 1:100;
2. OCT2: (Alexa Fluor 488) Anti-Oct-2 antibody (Abcam, UK), 1: 100;
3. N-cadherin: Anti-N-Cadherin antibody (Abcam, UK), 1: 200;
4. SGLT2: Anti-SGLT2 antibody (Abcam, UK), 1:200;
5. MRP4: anti-MRP4 antibody (Abcam, UK), 1:50;
6. MATE1: anti-MATE-1 antibody (Abcam, UK), 1:50;
7. OAT1: Anti-SLC22A6 antibody (Abcam, UK), 1:200;
8. OAT3: Anti-OAT3 antibody (Abcam, UK), 1:2000.

After incubation, the cells were washed three times with PBS for 5 min. Incubation of cells with the secondary antibodies was performed in the dark at room temperature for 1 h. The dilutions of the secondary antibodies were as follows:

1. N-cadherin: Goat anti-mouse IgG H&L (Alexa Fluor 488) preadsorbed ab150117 (Abcam, UK), 1: 500;
2. SGLT2, MRP4, OAT1, OAT3: Goat anti-rabbit IgG H&L (Alexa Fluor 488) ab150077 (Abcam, UK), 1: 500;
3. MATE1: Donkey anti-goat IgG H&L (Alexa Fluor 488) ab150129 (Abcam, UK), 1:500.

After incubation, the cells were washed three times with PBS for 5 min. Finally, two drops of the Mounting Medium Fluoroshield with DAPI were added, and the solution was left on the cells for 5 min. Micrographs were taken at the suitable wavelengths for respective dyes (excitation/emission: P-gp 652/668 nm, all others 495/519 nm).

Supplement S3.3. Staining for Podocyte (Negative for PTEC) Markers

Following the protocol for immunocytochemistry described in the methods section, the cells were incubated for 30 min with PBS, supplemented with 1% BSA and 0.1% solution of Tween 20 to block the non-specific binding of antibodies for primary antibodies. Incubation for 30 min with PBS supplemented only with 1% BSA was used for secondary antibodies. All incubations with the primary antibodies were performed overnight at 4°C. The dilution of the primary antibodies was as follows:

1. Podocin: Anti-NPHS2 antibody (Abcam, UK), 1:250.

After incubation, the cells were washed three times with PBS for 5 min. Incubation of cells with the secondary antibodies was performed in the dark at room temperature for 1 h. The dilution of the secondary antibodies was as follows:

1. Podocin: Goat anti-rabbit IgG H&L (Alexa Fluor 488) ab150077 (Abcam, UK), 1: 500.

After incubation, the cells were washed three times with PBS for 5 min. Finally, two drops of the Mounting Medium Fluoroshield with DAPI were added, and the solution was left on the cells for 5 min. Micrographs were taken at the suitable wavelengths for respective dyes (excitation/emission: 495/519 nm).

Supplement S4. Cell Polarisation

We chose two apical markers, P-gp and MATE-1, and two basolateral markers, OCT-2 and MRP-4 to show cell polarisation. Additionally, we stained the cells with epithelial lineage markers CK-18 and ZO-1. Cells were seeded and cultured on Transwell inserts at a density of 40.000 cells / well. We used a polycarbonate membrane (Corning). Fixation of cells was performed using the 4% formaldehyde for 10 min at room temperature, followed by washing the cells three times with PBS for 5 min. For staining, we used dilutions of the following solutions:

1) CK-18, AF488, 1:1000, dilution in PBS with added 1 % BSA and 0.1 % Tween 20;
2) ZO-1, FITC, 1:100, dilution in PBS with added 1 % BSA and 0.1 % Tween 20;
3) OCT2, P-gp, MATE1, MRP4 as described above.

Cells were permeabilized with a 0.1% solution of Triton X-100 in PBS for 10 min, followed by washing the cells three times with PBS for 5 min and blocking for 30 min with PBS with added 1 % BSA and 0.1 % Tween 20. All solutions were added in a ratio of 100 μL apical and 500 μL basolateral, and all incubations were performed overnight at 4°C. The next day, the cells were washed three times with PBS for 5 min, and secondary antibodies were added for one hour at room temperature as described above, followed by washing the cells three times with PBS for 5 min. Finally, two drops of the Mounting Medium Fluoroshield with DAPI were added, and the solution was left on the cells for 5 min.

Supplement S5. Protocol for Confocal Microscopy Used in Polarisation Experiments

Inserts containing the cell lines were trimmed of plastic walls and transferred to the imaging chamber of a LEICA SP8 Stellaris upright confocal system (HC APO L U-V-I 63x/0.90 WATER) (Leica Microsystems, Germany) filled with physiological saline solution and fixed using custom made weight. We resorted to sequential imaging between DAPI and other dyes to minimize crosstalk. DAPI staining was imaged using excitation laser line 405 nm and emission light was collected using an HyD S detector (counting mode) in the range 420nm – 720 nm (512 x 512 pixels @ 700 Hz, line accumulation 3-16, frame average 1-8, pinhole 1 AU). ZO-1 staining was imaged using excitation laser line 488 nm, and emission light was collected using an HyD X detector (counting mode) in the range 500 – 740 nm (512 x 512 pixels @ 700 Hz, line accumulation 16, frame accumulation 4, pinhole 1,5 AU). Cytokeratin-, MPR-4-, and OCT-2-stained cells were imaged using excitation laser line 498 nm, and emission light was collected using an HyD X detector (counting mode) in the range 508 - 740 (512 x 512 pixels @ 700 Hz, line accumulation 14-16, frame accumulation 4-6, pinhole 1 – 1,3 AU). Mate-1 staining was imaged using excitation laser line 500 nm, and emission light was collected using an HyD X detector (counting mode) in the range 510 – 740 nm (512 x 512 pixels @ 700 Hz, line accumulation 16, pinhole 1,3 AU). Phalloidin staining was imaged using excitation laser line 553 nm, and emission light was collected using an HyD X detector (counting mode) in the range 558 – 740 nm (512 x 512 pixels @ 700 Hz, line accumulation 8, frame accumulation 2, pinhole 1,3 AU). P-glycoprotein staining was imaged using excitation laser line 644 nm, and emission light was

collected using an HyD X detector (counting mode) in the range 657 – 740 nm (512 x 512 pixels @ 700 Hz, line accumulation 16, frame accumulation 2, pinhole 1,2 AU).

Supplement S6. Figures

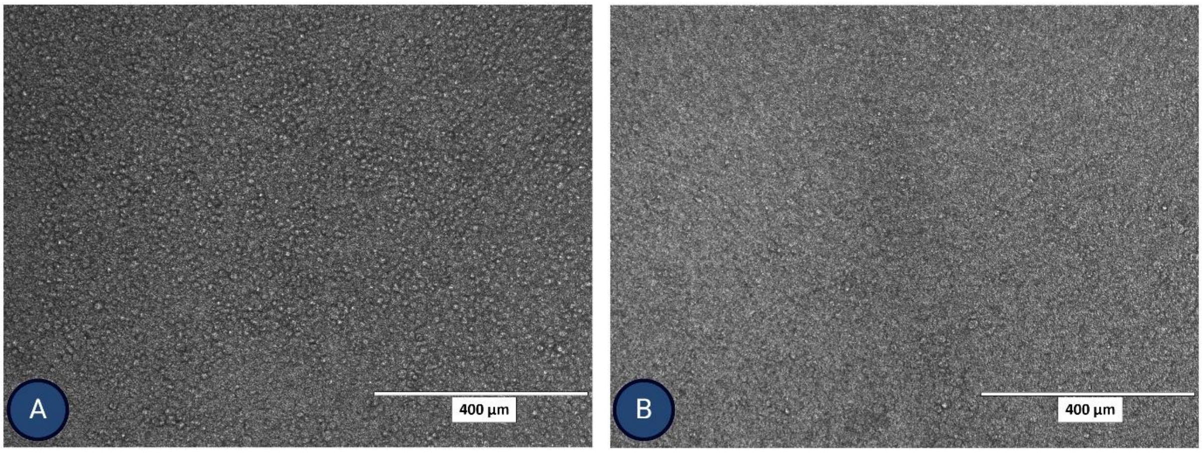

Figure S1. Micrographs of the reached confluent cell culture grown on inserts using protocols 1 (A) and 2 (B). The magnification was 10× (according to the manufacturer's microscope specifications, EVOS FL Cell Imaging System, Thermo Fisher Scientific, USA). The micrographs appear noisy due to the polycarbonate membrane.

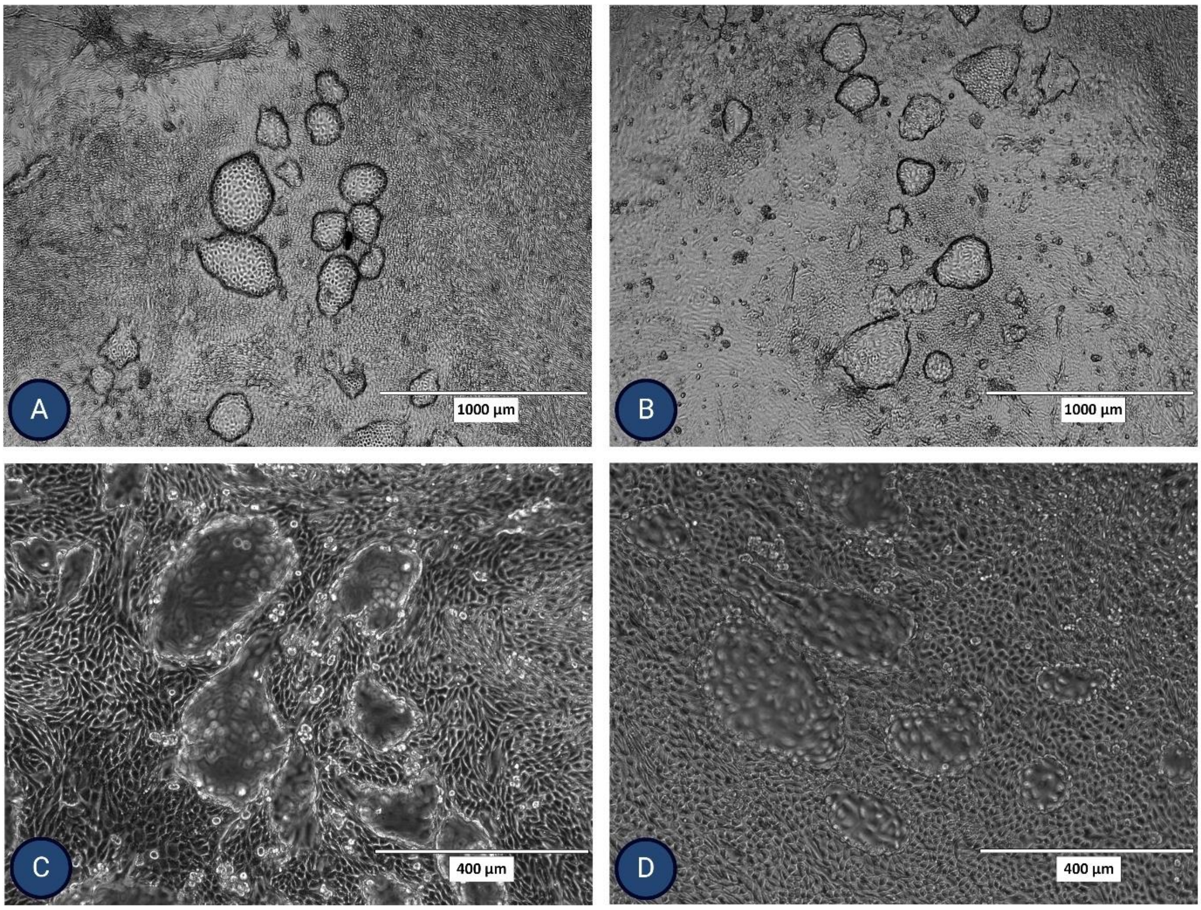

Figure S2. Micrographs of the formed hemicysts for cells isolated using protocols 1 (A and C) and 2 (B and D). The magnification was either 4× (A and B) or 10× (B, C, and D) (according to the manufacturer's microscope specifications, EVOS FL Cell Imaging System, Thermo Fisher Scientific, USA).

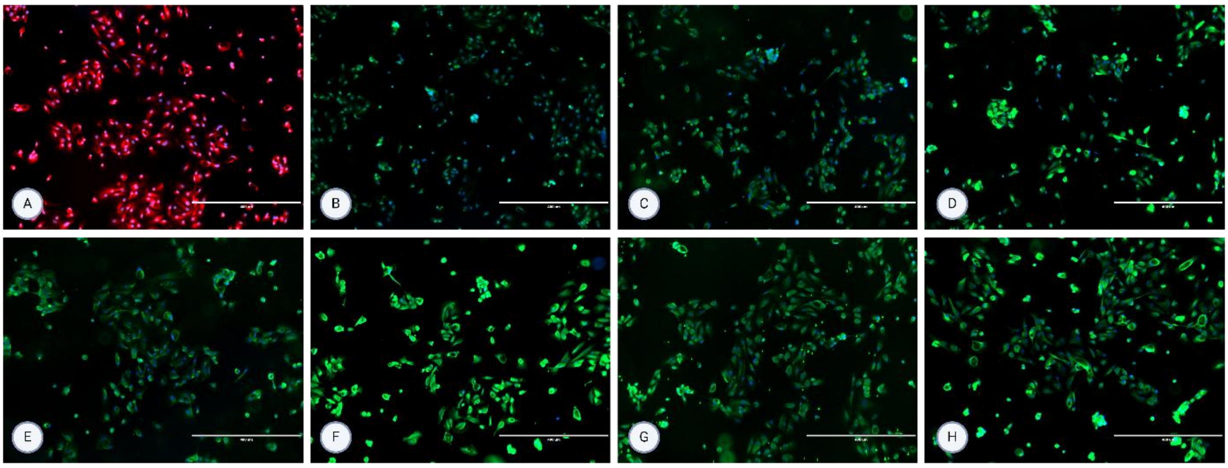

Figure S3. Micrographs of the stained samples in the third passage following protocol 1: (Supp1A) for P-gp, (Supp1B) OCT2, (Supp1C) N-cadherin, (Supp1D) SGLT-2, (Supp1E) MRP4, (Supp1F) MATE1, (Supp1G) OAT1, and (Supp1H) OAT3. For all samples, a mounting medium with DAPI was used to stain the nuclei. The magnification of all shown images is 10× (according to the manufacturer's microscope specifications, EVOS FL Cell Imaging System, Thermo Fisher Scientific, USA).

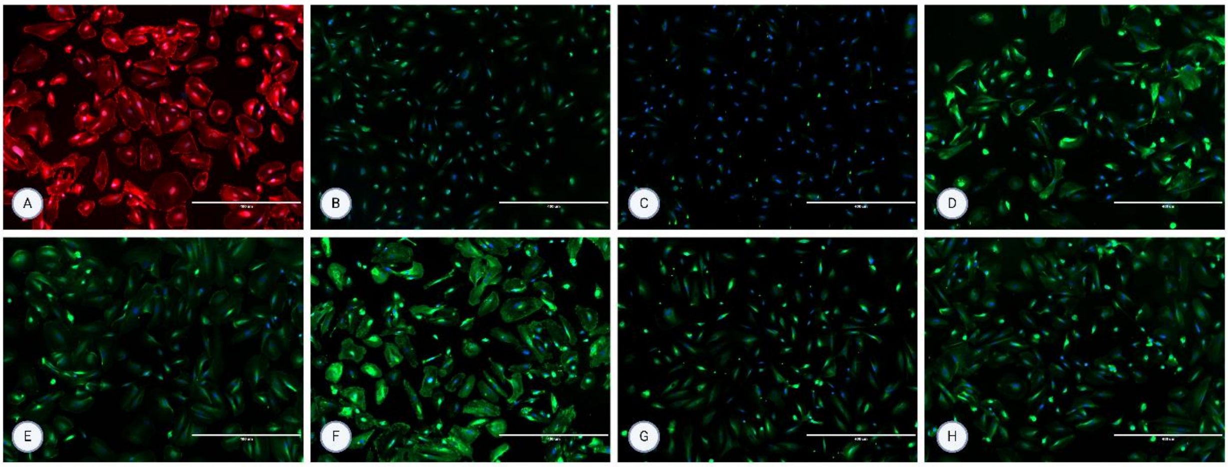

Figure S4. Micrographs of the stained samples in the third passage following protocol 2: (Supp2A) for P-gp, (Supp2B) OCT2, (Supp2C) N-cadherin, (Supp2D) SGLT-2, (Supp2E) MRP4, (Supp2F) MATE1, (Supp2G) OAT1, and (Supp2H) OAT3. For all samples, a mounting medium with DAPI was used to stain the nuclei. The magnification of all shown images is 10× (according to the manufacturer's microscope specifications, EVOS FL Cell Imaging System, Thermo Fisher Scientific, USA).

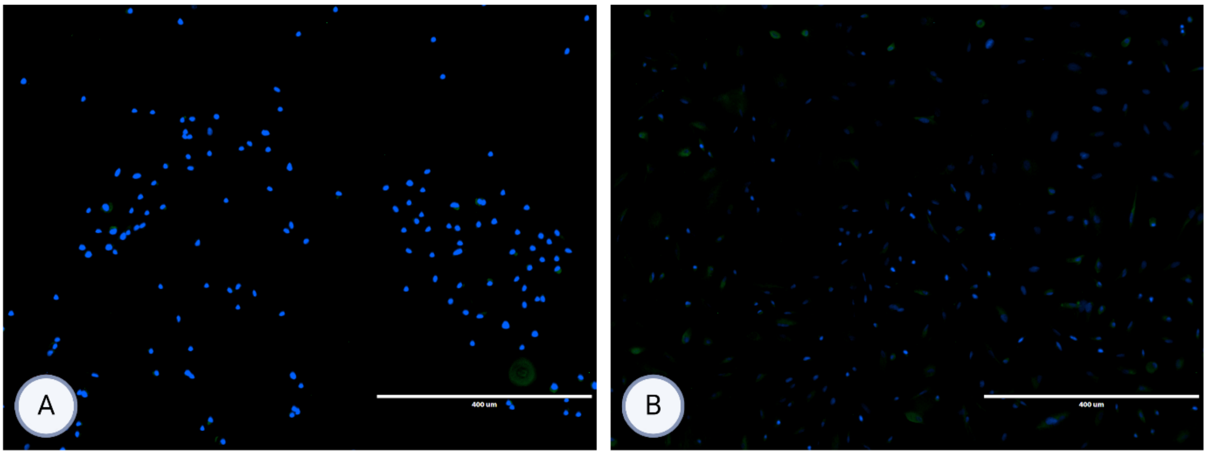

Figure S5. Micrographs of the podocin-stained (green) samples in the third passage following protocols 1 (A) and 2 (B). For all samples, a mounting medium with DAPI was used to stain the nuclei. The magnification of all shown images is 10× (according to the manufacturer's microscope specifications, EVOS FL Cell Imaging System, Thermo Fisher Scientific, USA).

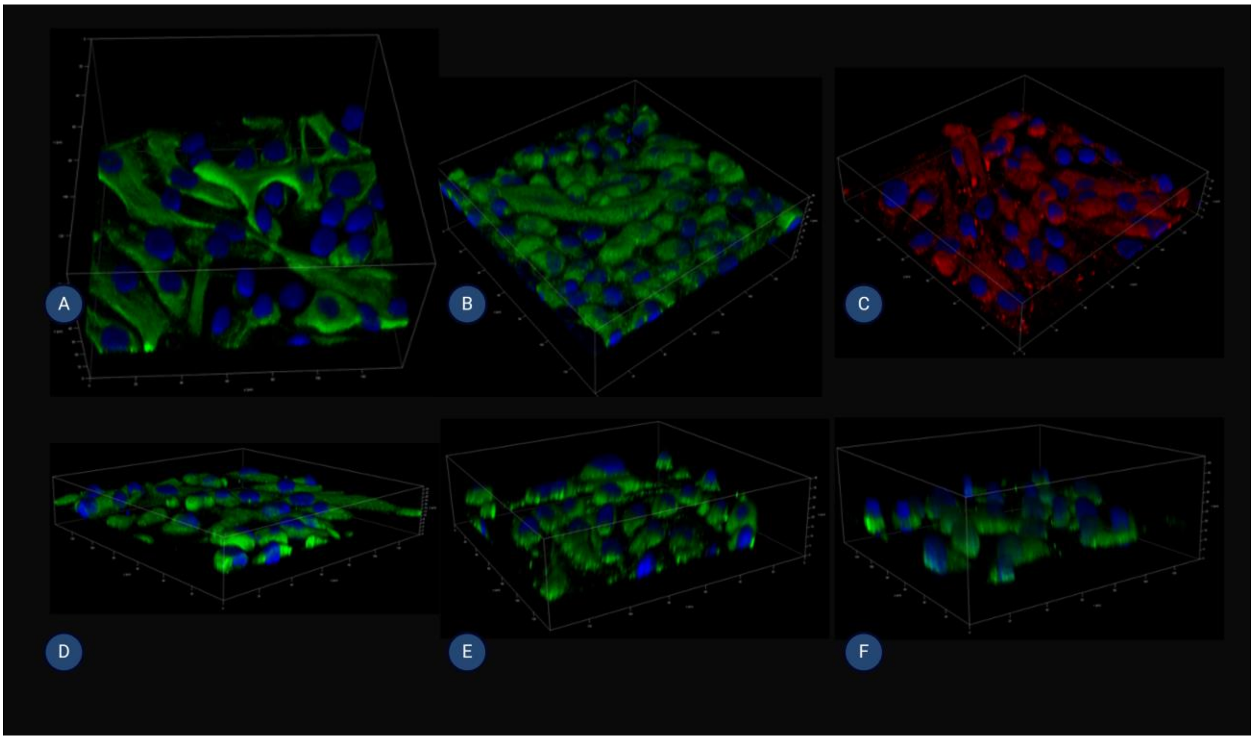

Figure S6. 3D micrographs of stained MFUM-RPTEC-1 grown on inserts in the first passage following protocol 1 shown in green and red. (Supp4A) for CK-18, (Supp4B) ZO-1, (Supp4C) P-gp, (Supp4D) MATE-1, (Supp4E) MRP4, and (Supp4F) OCT2. A mounting medium with DAPI was used to stain the nuclei (shown in blue) for all samples. The magnification of all images shown is 63× (according to the manufacturer's microscope specifications, LEICA SP8 Stellaris upright confocal system, Leica Microsystems, Germany).

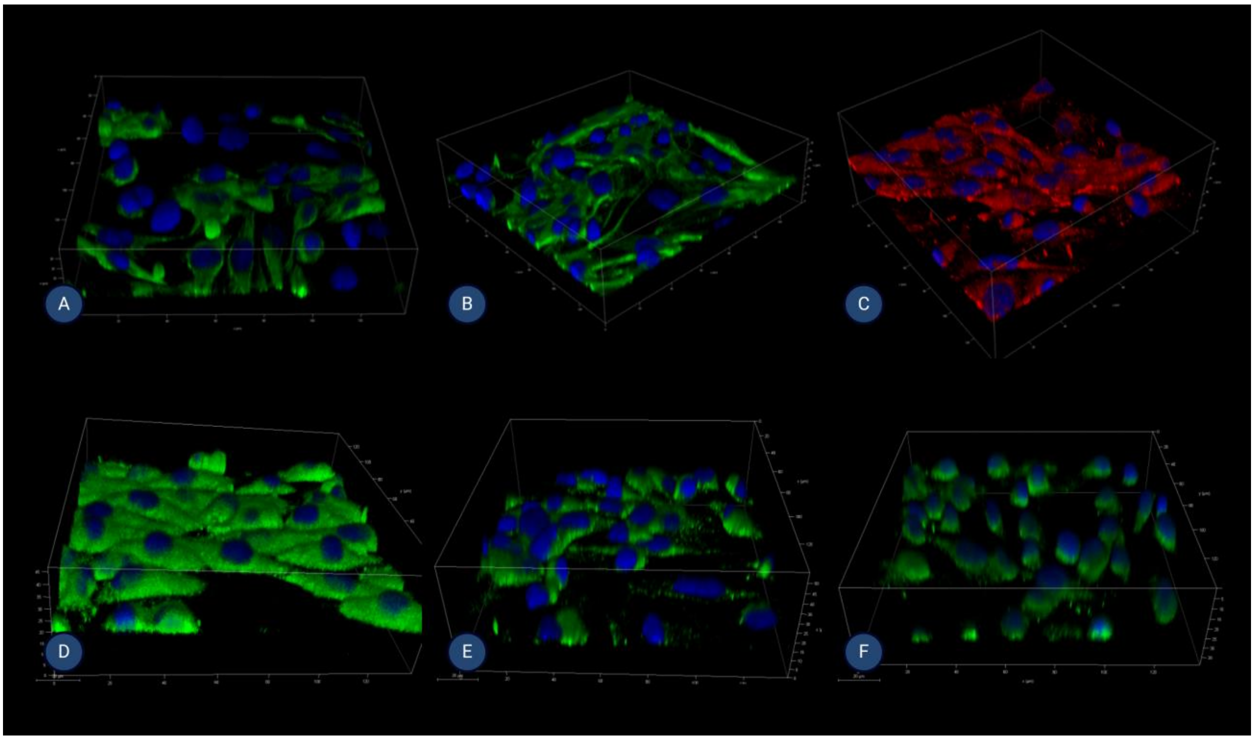

Figure S7. 3D micrographs of stained MFUM-RPTEC-2 grown on inserts in the first passage following protocol 2 shown in green and red. (Supp5A) for CK-18, (Supp5B) phalloidin, (Supp5C) P-gp, (Supp5D) MATE-1, (Supp5E) MRP4, and (Supp5F) OCT2. A mounting medium with DAPI was used to stain the nuclei (shown in blue) for all samples. The magnification of all images shown is 63× (according to the manufacturer's microscope specifications, LEICA SP8 Stellaris upright confocal system, Leica Microsystems, Germany).

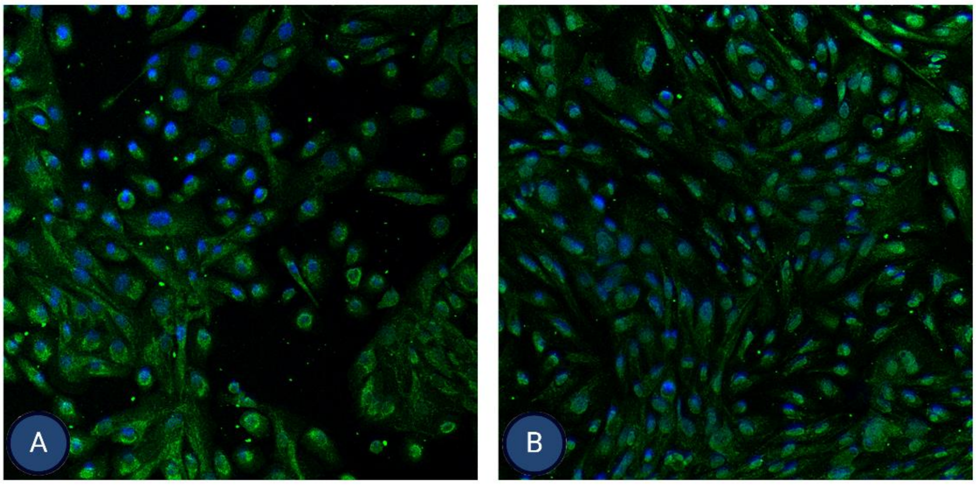

Figure S8. Micrographs of stained MFUM-RPTEC-1 (A) and MFUM-RPTEC-2 (B) grown on inserts for ZO-1. A mounting medium with DAPI was used to stain the nuclei (shown in blue) for all samples. The magnification of both images shown is 63× (according to the manufacturer's microscope specifications, LEICA SP8 Stellaris upright confocal system, Leica Microsystems, Germany).
